# Androgenic-Anabolic Steroids: From the Gym to Drug-Induced Liver Injury

**DOI:** 10.7759/cureus.28798

**Published:** 2022-09-05

**Authors:** Ana Sofia Alves, Sofia Perdigão, Sandra Morais, Cristiana Sousa, Fernando Salvador

**Affiliations:** 1 Internal Medicine, Centro Hospitalar de Trás os Montes e Alto Douro, Chaves, PRT; 2 Internal Medicine, Centro Hospitalar de Trás os Montes e Alto Douro, Vila Real, PRT

**Keywords:** drug induced liver injury, cytocholestasis, liver toxicity, liver injury, androgenic-anabolic steroids

## Abstract

Drug-induced liver injury (DILI) is a frequent cause of liver toxicity. We describe the case of a 32-year-old male patient without any relevant past medical history or medication use. In the past two months, he was engaged in weightlifting exercises and consumed androgenic-anabolic steroids to enhance his exercise routine. The patient initially experienced choluria and acholia for two weeks, followed by itching for two days, which led him to present to the emergency room. His laboratory results revealed cytocholestasis. He was admitted for investigations and after excluding other causes of liver injury, the diagnosis of DILI related to the consumption of androgenic-anabolic steroids was made. This case report highlights the perils of using performance-enhancing substances such as androgenic-anabolic steroids, which may lead to severe side effects like DILI.

## Introduction

Drug-induced liver injury (DILI) is one of the most common causes of drug withdrawal from markets currently [1-4]. The incidence of DILI is about 10.15 per 100,000 individuals [5], and it accounts for around 10% of all cases of acute hepatitis; it is also the most frequent cause of acute liver failure in the United States (US) [6-8]. In the US, the drugs more frequently associated with DILI are acetaminophen and the combination of amoxicillin and clavulanic acid [9-11].

Androgenic-anabolic steroids can be natural or synthetic products and are used to gain muscle mass and lose body fat. As per a meta-analysis of 187 studies, the prevalence of androgenic-anabolic steroid consumption was reportedly 3.3%, and the majority of cases involved non-professional athletes, which points to a dangerous reality and a serious public health issue [12-13].

We present the case of a previously healthy male who developed DILI after the consumption of androgenic-anabolic steroids.

## Case presentation

The patient was a 32-year-old male, a baker, who did not have any relevant past medical history. There was no family history of regular medication use, and no consumption of alcohol, tobacco, or any drug abuse.

The patient's laboratory tests showed hyperbilirubinemia caused by direct bilirubin (DBrb), cytocholestasis, and dyslipidemia (elevated LDL cholesterol and triglycerides); he was subsequently sent to the emergency room. He presented with jaundice, choluria, and acholia for two weeks and itching for two days. There was no history of tea and wild mushroom consumption, or any risky sexual contacts. After an exhaustive anamnesis, he admitted to consuming synthetic androgenic-anabolic steroids (2,3-epithio-17 a-methyl-17b-hydroxy-5y androsterone). His physical exam revealed jaundice. The blood tests at the emergency room revealed eosinophilia (490/uL; normal value: 60-460) and elevated aspartate aminotransferase (AST) of 84 U/L (normal value: <40), alanine aminotransferase (ALT) at 138 U/L (normal value: <41), lactic dehydrogenase (LDH) at 258 U/L (normal value: 135-225), total bilirubin (TBrb) at 9.40 mg/dl (normal value: <1.2), DBrb at 9.00 mg/dl (normal value: <0.3), total proteins at 7.9 g/dl (normal value: 6.6-8.7), albumin of 5.2 g/dl (normal value: 3.4-4.8), and no alteration on coagulation tests. The abdominal CT revealed moderate hepatomegaly, slight splenomegaly, and no dilation or obstruction of bile ducts. In light of this, the patient was admitted for investigations of cholestatic hepatitis.

**Figure 1:**
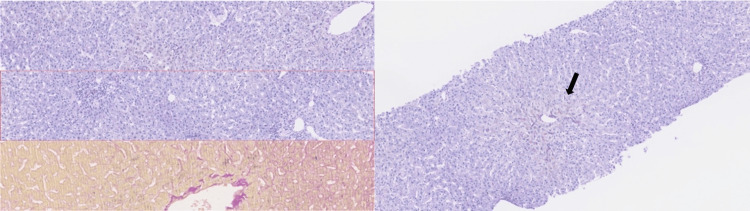
Liver biopsy Histology with aspects of acute hepatitis on high resolution

During his hospital stay, he experienced liver dysfunction: hyperbilirubinemia (maximum value of 26 mg/dl) caused by DBrb (maximum value of 21 mg/dl), and also impairment related to total cholesterol (maximum value of 385 mg/dl) and LDL cholesterol (maximum value of 321 mg/dl); the patient also had a decrease of HDL cholesterol (minimum value of 4 mg/dl). The remaining blood tests - serological tests (HIV; hepatitis A, B, C; Epstein-Barr; cytomegalovirus; infectious mononucleosis and brucellosis) and autoimmune tests (ANA, anti-mitochondrial, anti-SLA, anti-LC1, and anti-smooth muscle antibodies) - were negative. After the exclusion of other etiologies of liver failure, a diagnosis of DILI was assumed based on his steroid abuse. At the same time, he was started on treatment with intravenous N acetylcysteine (NAC) protocol (NAC 150 mg/kg in one hour, followed by NAC 50 mg/Kg in four hours, and then NAC 100 mg/kg in 16 hours) and oral corticoids with prednisolone 1 mg/kg/day. A liver biopsy was also performed, with the histological findings of canicular cholestasis injuries, which were suggestive of hepatitis on the high-resolution image (black arrow), as shown in Figure 1.

Contrary to the expectations at the beginning of the treatment, the patient's condition did not improve, and hence he underwent a pre-transplant study, due to the unpredictability of the clinical course.

After one month of management, including symptomatic treatment, the patient was discharged under ursodeoxycholic acid (UDCA) for itching, the use of which is not well-documented in the literature, but led to favorable results in our patient. He was under strict monitoring during the ambulatory consult and subsequently at the hepatology consult. He experienced progressive improvement of hepatic function with total normalization after three months.

## Discussion

Even though DILI is a rare disease, it remains one of the most frequent causes of drug withdrawal from the market. It can also lead to serious adverse effects in some cases [1,2,8]. DILI can be classified into different subtypes according to its physiological mechanisms: cytotoxic (associated with hepatocellular injury), cholestatic, or mixed. It can also be classified into acute, if the duration is less than three months, or chronic, if the duration is more than three months [2,14,15]. Chronic DILI has clinical manifestations that can be similar to chronic hepatic liver disease, autoimmune hepatitis, primary biliary cirrhosis, and non-alcoholic fatty liver disease (NAFLD) [2,3]. DILI's clinical manifestations can range from asymptomatic, with a slight increase in aminotransferases, to symptomatic forms: with severe cholestasis and itching, acute hepatitis and jaundice, or acute liver failure [1,6-7,14,16-17].

Cholestatic DILI is defined as an increase in alkaline phosphatase (ALP) greater than two times the normal value and/or an ALT/ALP ratio of less than 2 [18]. The use of androgenic-anabolic steroids is usually associated with cholestatic DILI, and it is also related to a decrease in HDL cholesterol and an increase in total and LDL cholesterol levels [12,13].

We discussed a case of cholestatic DILI induced by the consumption of synthetic androgenic-anabolic steroids, with an increase in aminotransferases and bilirubin. As per Hy’s Law, having an ALT level three times greater than normal plus a serum bilirubin level two times greater than normal is associated with a bad prognosis [19,20]. Our patient fulfilled the criteria for a poor prognosis: his ALT level was three times the upper level of normal and his serum bilirubin level was eight times the upper level of normal. Hence, a pre-transplant study was performed, even though the patient did not have hepatic encephalopathy and/or coagulopathy, which are considered signs of acute liver failure. The patient was on follow-up for one year with hepatology, and he was found to maintain normal values of bilirubin and aminotransferase. He was discharged with recommendations to avoid androgenic-anabolic steroids.

The 17α-alkylated androgenic-anabolic steroids are popular among athletes due to their ease of oral uptake; examples include testosterone, stanozolol, or nandrolone. The abusive consumption of these substances can lead to severe adverse side effects, such as an increase in thrombotic risk, and cardiovascular risk with dyslipidemia [12,13]. Also, it has been shown that this kind of androgenic-anabolic steroids can induce liver toxicity by way of direct mechanisms. This case report sheds light on the serious adverse effects of androgenic-anabolic steroids when used for recreational purposes or for performance enhancement.

Table 1 presents a literature review of DILI.

**Table 1:** Literature review of drug-induced liver injury

Article	Article/study type	Evidence
Friis and Andreasen, 1992 [6]	Population-based study	This study reviewed 1,100 reports of adverse drug reactions associated with DILI and concluded that paracetamol was reported to induce acute cytotoxic as well as cholestatic reactions in non-alcoholic subjects taking therapeutic doses
Sgro et al., 2002 [5]	Population-based study	The study assessed the incidence and gravity of hepatic injury in adverse drug reactions over a period of 3 years; 34 cases were reported in a French population. It was concluded that the incidence and seriousness of drug-induced hepatitis are largely underestimated in the general population; in this particular study, the incidence of hepatic injury was 16 times higher than anticipated by spontaneous reports
Ostapowicz et al., 2002 [9]	Prospective study	The study analyzed 17 tertiary care centers in the United States with an aim to examine the clinical features, presumed causes, and short-term outcomes of acute liver failure; it concluded that acetaminophen overdose and idiosyncratic drug reactions have replaced viral hepatitis as the most frequent apparent causes of acute liver failure
Chang et al., 2007 [4]	Review article	This article reviewed the hepatotoxicity of select commonly used drug classes, including anabolic steroids. It concluded that amoxicillin-clavulanic acid is one of the most frequently implicated causes of drug-induced liver injury worldwide, followed by statins and antiretroviral drugs. It also concluded that genetic polymorphisms may account for some of the differences in individual susceptibility to drug hepatotoxicity
Chalasani et al., 2008 [7]	Prospective study	This study included 300 patients and concluded that DILI in the United States is caused by a wide variety of prescription and nonprescription medications, nutritional supplements, and herbal supplements, with antibiotics accounting for the majority of the cases (45%). It also concluded that 20% of cases of DILI were caused by more than one hepatotoxic agent
Barceloux et al., 2013 [12]	Original article	The article reviews the characteristics of anabolic steroids, the epidemiology of their use, their dose-dependent effects, toxicity, and mechanisms of action, and elaborates on treatment methods, which are essentially supportive

The diagnosis of DILI can be difficult and it is an underdiagnosed condition; we made our diagnosis by ruling out other possible entities. Our patient was seriously ill and presented with typical manifestations of liver toxicity. A diagnosis of cholestatic DILI was made, based on several severe disease markers. We initiated and continued with the required treatment, which led to an improvement in the patient's condition.

## Conclusions

The recreational use of androgenic-anabolic steroids, usually by non-professional athletes, is a growing phenomenon and a serious public health issue. At the same time, DILI remains the principal reason for drug withdrawal from the market and it is also the cause of acute hepatitis in 10% of the cases. Also, it is critical to be alert to the liver toxicity that these substances can induce, and early and prompt detection of any liver injury is paramount to avoid the development of DILI. In some cases, there may be discrepancies between initial clinical manifestations and laboratory results. DILI in severe cases can progress to acute liver failure and is an indication for liver transplantation, which further emphasizes the importance of early diagnosis and treatment. Primary care clinics should raise awareness among people about the perils of using anabolic steroids so that severe side effects like DILI can be effectively prevented.

Our patient, in spite of the severity of his condition, had a favorable outcome and did not need to undergo liver transplantation. Also, another interesting aspect of this case was the use of UDCA for symptomatic control, which helped in the overall improvement of our patient; however, this association is not well described in the literature.
